# Thrombectomy for Stroke in Brazil—Late Evidence or Promising Future?

**DOI:** 10.3389/fsurg.2021.651183

**Published:** 2021-04-28

**Authors:** Pedro Tadao Hamamoto Filho, Gabriel Pinheiro Módolo, Carlos Clayton Macedo de Freitas, Marco Antônio Zanini, Rodrigo Bazan

**Affiliations:** Department of Neurology, Psychology and Psychiatry, Botucatu Medical School, UNESP – Univ Estadual Paulista, Botucatu, Brazil

**Keywords:** thrombectomy, stroke, trials of treatment, health systems, community

Martins et al. recently published a trial titled “Thrombectomy for Stroke in the Public Health Care System in Brazil”—the RESILIENT trial—in which they randomized 111 patients to receive standard care plus thrombectomy and 110 patients in the control group (standard care alone) (1). Like other trials that have been published since 2015, the authors found that endovascular treatment within 8 h after stroke onset provides better functional outcomes than standard care alone (2–4).

One may argue that the results of the trial are not new and, maybe, it was unethical to randomize patients not to receive thrombectomy. However, this study was necessary to show the feasibility of thrombectomy for stroke in a middle-income country. Actually, this is a welcome study that may foster public health managers to approve the routine use of this treatment modality, which could save near 2 million lives a year if expanded globally (5).

This challenge is a worldwide concern, which led the Society of Vascular and Interventional Neurology to launch the Mission Thrombectomy, aiming for a global expanding of thrombectomy to reduce the morbidity and mortality related to stroke. Despite this effort, many organizational restrictions persist, keeping important inequalities and disparities in the access to thrombectomy (6).

The Brazilian health system (SUS) is based on the principle of health as a citizen's right and the state's duty; it therefore relies on the doctrinal principles of universality, integrality, and equity (7). For instance, these principles have led the country to experience a successful response to HIV infection, with a policy of universal availability of highly active anti-retroviral therapy since 1996 (8).

Fortunately, the accessibility for stroke care has also improved over the last decade, mainly due to the efforts by the National Stroke Policy Act, which defined the requirements and levels of stroke centers, improved the specific budget for stroke care and rehabilitation, and helped funding training health professionals for stroke interventions (9). However, the continental dimensions and the large disparities between the different regions of the country keep the population access for stroke treatment still heterogeneous. The majority of stroke centers are located in capital and large cities, and therefore, the inner parts of the country, as well as rural areas, still lack the gold standard treatment.

With regard to thrombectomy for stroke, the concern will be how to provide universal access to high-tech treatment in a country with such high disparities. The RESILIENT investigators themselves recognize that most of the treating hospitals had only one or two angiography suites for multiple specialties. Besides, different centers contributed different numbers of patients; this demonstrates heterogeneity, even in a controlled setting.

It took 17 years from the National Institute of Neurological Disorders and Stroke rt-PA Stroke Study Group (NINDS) (10) trial publication to the Brazil government's approval of alteplase in the SUS. Based on this, one can imagine the length of time it may take for the Brazilian government to approve thrombectomy, even after a national powerful trial. Nevertheless, it is important to highlight that the Brazilian Ministry of Health supported the trial, and despite the high costs of thrombectomy, its efficacy and cost-effectiveness are proven for a limited resource setting.

Future political and research initiatives should explore ways to expand the accessibility to thrombectomy and reduce its costs. A possible way to accelerate the time from clinical onset of stroke to the endovascular thrombectomy would be exploring the safety and efficacy of treatment under local anesthesia. In low- and middle-income countries, this would allow for wider availability without the constraint related to the routine use of general anesthesia in both adult (11) and pediatric population (12). Other initiatives, such as task sharing in neurosurgery (that is, delegating certain neurosurgical tasks to non-neurosurgical specialists) and partnerships between developing countries for international training programs, could be useful to increase neurosurgical capacities in regions where workforce deficit remains substantial (13, 14).

In summary, the RESILIENT trial opens the door for a promising future for stroke care in low- and middle-income countries. However, this future will depend on the translation of scientific evidence to public policies. The scientific community did its part.

## Author Contributions

PH, CM, and RB: conception. GM, MZ, and RB: analysis of data. PH: draft. GM, CM, MZ, and RB: critical review. All authors have final approval of the manuscript and agreement to be accountable for all aspects of the work.

## Conflict of Interest

The authors declare that the research was conducted in the absence of any commercial or financial relationships that could be construed as a potential conflict of interest.
